# Abstracts from German Journals

**Published:** 1872-06

**Authors:** Ch. Raushenberg

**Affiliations:** Atlanta, Georgia


### ABSTRACTS FROM GERMAN JOURNALS.

BY CH. RAUSHENBERG, M.D., ATLANTA, GEORGIA.

*HYDROPS GENU INTERMITTENS. By Dr. Paul Bruns, Assistanl Physician to tne Surgical Clinic, Tuebingen, Germany.*

The interesting phenomenon of an exquisitely intermittent hypropical effusion into the knee-joint; its spontaneous appearance and disappearance, occupying each time a regular period of eleven days; the permanency of the disease, and its peculiar congruity with the case of Dr. Löwenthal, from the Heidelberg Clinic—the first one of the kind in the literature of medicine, so far as the author is able to ascertain—published in No. 48 of the *Berliner Klinik Woehenshrift*, of 1871; and the interest connected with a physiological interpretation of the origin and nature of these cases, caused Dr. Bruns to publish his case, and the translator to give the following short reproduction of its leading features:

Marie Ecker, servant girl, unmarried, twenty-eight years old, entered the clinic in August, 1853; has been healthy up to her twentieth year. Late sexual development; first appearance of menstruation at twenty-four; has been regular since, without pains, but scanty, and of short duration. At the age of twenty, patient was taken the first time with an affection of the knee. Considerable tumefaction and pain in the left knee-joint, without any local redness or heat, but with rigors, headache, febrile excitement, followed by perspiration, made their appearance suddenly, without any perceptible external cause, and without any gastric disturbances.

The tumefaction, with the other phenomena, disappeared spontaneously after a duration of six days; patient remained completely well five days, and on the twelfth day a paroxysm of exactly the same character recurred. These attacks continued with the utmost precision, every twelfth day, for a period of eight years. The general febrile symptoms and the pain in the diseased joint, after a few paroxysms, failed to reappear, and thus the disease very soon presented simply the appearance of *hydrops genu intermittens.* It is remarkable, however, that these periodical effusions for awhile affected both knees.

The patient was, during the whole duration of her disease, either employed as a servant girl or under treatment for weeks and months at the hospitals of Pforzheim and Carlsruhe, and the clinics of Heidelberg and Freiburg. Vesicatories, local douches, steam baths, the mineral waters of Wildbad, etc., led to very unsatisfactory results; and only the internal use of quinine, and vesicatories, at the clinic at Heidelberg, is said to have effected temporary cessation of the effusions, and a diminution of their quantity.

Patient remained at the clinic at Tuebingen from August, 1853, until February, 1854. She is described as not very robust, but well nourished and of good constitution, with good appetite and digestion, but tardy stools. Action of her heart normal; menstruation regular, but scanty, and only lasting thirty hours. She was, for the sake of accurate observation, kept in bed some time, without therapeutical interference of any kind. The diary shows the following characteristics of her case:

A spontaneous swelling of the knee-joint occurs regularly every twelve days, each paroxysm occupping six days, and being followed by an intermission of five days. For instance, in one month we find in the diary: "Attack from 2d to 7th, intermission from 8th to 12th; attack from 13th to 18th, intermission from 19th to 23d; attack from 24th to 29th," etc. No prodromi to the paroxysms, except a peculiar indistinct individual sensation, each paroxysm presenting a distinct stadium incrementi, acmes and decrementi. The swelling of the joint increases in two days from thirty-seven to forty-two centimeters; remains stationary one day, and decreases in size three days, until it reaches the normal size on the sixth day.

The objective phenomena during the paroxysms are simply those of serous intra-capsular effusion of the left knee-joint, without any inflammatory symptoms; patella in normal situation under the skin; no effusion into the bursa patellaris; the joint not painful on pressure, nor under active or passive motion—a local sensation of heaviness and pressure being the only individual symptoms; patient is able to perform light labor; no functional disturbances, no increase of bodily temperature; the menstruation exercises no influence on the appearance and course of the attacks—they occur regularly during its existence; patient is hardy, perfectly able to work during the intervals, and the joint does not exhibit the slightest appearance of effusion, but looks exactly like the sound knee.

The result of the treatment instituted during the patient's stay at the clinic at Tuebingen was as follows:

1. Efforts to prevent the appearance or reduce the tumefaction of the knee-joint, by a pressure-bandage, were utterly unavailable.

2. The internal use of quinine, from nine to thirty grains daily, for a period of six weeks, reduced the degree of the swelling slightly, and shortened the duration of the paroxysms, and hence lengthened that of the intermission two days; but the eleven-day type continued. The application of the hot iron, in stripes along both sides of the spine, furnished no distinct result.

3. The internal use of Fowler's solution, with tincture chinoid (℞—Solut. arsen. Fowl. grs. 18 to ʒ i; tinct. chinoid ℥ i), in doses of a tea-spoonful thrice daily, had a strikingly favorable effect. The paroxysms lost their intensity and duration, and after using Fowler's solution in doses of from six to twenty drops, the patient was dismissed in February, 1872.

The duration of this cure lasted twenty months; the disease then returned, affecting first the right, and, after two paroxysms, both knee-joints, and assuming the former eleven-day type. The swelling of the formerly diseased joint appeared generally two days later than in the right knee-joint; reached its point of culmination, 37 centimeters* circumference, at the same time with the latter, which generally attained 42.5 centimeters circumference, and disappeared at the same time. Four weeks' treatment with solut. arsen. Fowleri had the same decided effect as before on the left; seven weeks' treatment on the right knee-joint. Patient was discharged in the summer of 1856, and her perfect recovery confirmed by her own statement nine months afterward.—*Berlin Wochenschrift, January,* 1871, *No.* 1.

* One inch about equal to 2½ centimeters.

### *PERFORATION OF THE UTERUS BY THE PROBE.*

In a meeting of the Obstetrical Society of Berlin, November 14th, 1871, Mr. Rabl-Bückhardt reported the case of a woman who was admitted into the Charité of that city four weeks after her last confinement. In repeated examinations of the uterus, the uterine probe entered sixteen and a half and nineteen centimeters,* respectively, without any hemorrhage or other reaction of importance following. As she died afterwards with tuberculosis, the probe was introduced during post-mortem examination, and entered to the same length. On inspection, the very vascular and almost cavernous fundus uteri showed one scar evidently the result of the former perforation, and one opening that of the last one. Four other cases were mentioned, where the probe was observed to enter the uterus from sixteen to twenty centimeters (thirty to one hundred and fifty days after post-partum.)

Mr. L. Meyer has seen one case, Mr. Martin several, of the same kind, particularly after long-continued amenorrhœa following delivery. Perforation of the uterus must be supposed to have occurred in all these cases, but no perceptible degree of reaction was observed in any of them.—*Berlin Klinik Wochenschrift, No.* 1, 1872.

---

### *INHALATION OF BROMIUM IN DIPHTHERITIC AND CROUPOUS PROCESSES.*

The well-established fact that croupous membranes are much easier soluble in bromo-bromide of potash than in lime-water, or any other substance, caused Schütz, some time ago, to resort to inhalations of bromium in this disease. His success has been so universal that he has repeatedly, and lately again (*Wiener Medicinische Wochenschrift*, Nos. 31, 32 and 33), called the attention of the profession to his method of treatment. He uses a solution of bromii pur, potassii bromidi, aa grm. 0.3 (4½ grs.); aq. distil. grm. 150.0 (4 ℥ s 5½ ʒ.) A sponge soaked in this solution, laying at the apex of a cone, formed of strong

*Two and a quarter centimeters nearly equal to one inch.

paper, is brought to the nose of the patient in the same way for inhalation as chloroform. This is done every half hour, or every hour, from five to ten minutes at a time. Children, even within the first year of life, bear these inhalations well.

The above preparation is very volatile, and easily decomposed under the influence of the light, and must, therefore, be kept tightly closed in a dark place. The free use of emetics, whenever the larynx is implicated, must be connected with this treatment.—*Berlin Klinik Wochenscrift, No.* 7, 1872.

---

"*SURGERY OF THE KIDNEYS*"

Is the title of a late German work, written by Dr. Gustav Simon, Professor of Surgery at Heidelberg; published by F. Enke, at Erlangen. Prof. Sieg. Rosenstein, of Groveningen, the distinguished author of a work on the diseases of the kidneys, notices this production in the following terms:

"Not only the title of this small volume is so far unheard of, but also its contents are materially new.

"Prof. Simon, after other efforts had failed, extirpated a perfectly healthy kidney, with complete success, for the cure of a uretero-abdominal fistula. If this small work contained but the description of the case under consideration—of the operation, its course, and after-treatment—we would have cause to be thankful for it; but it becomes much more valuable, as it contains the studies and experiments of the author, which justified him in his bold operative interference. Simon has not operated, trusting to good luck, or on the basis of vague surmises; but only after he had theoretically and practically, and with exhausting thoroughness, and in their most extensive bearing, considered all questions connected with the subject. The influence which the loss of one functionally unimpaired kidney must have upon the other—whether a fear of the uræmic infection of the blood is to be entertained—whether by the sudden extinction of so large a tributary of the capillaries, a secondary hypertrophy of the heart can be brought on—all these questions have been experimentally decided before the operation; and it affords, indeed, a moral satisfaction to see the surgeon so deeply engaged in the solution of difficult pathological questions before he resorts to the knife.

"I confirm, with particular satisfaction, that my own experiments on these questions (*Virchow's Archiv.*, Vol. 53), in all their results, with the exception of very immaterial differences, agree fully with those of Simon. * * * A new track for the treatment of some kidney diseases, considered entirely unapproachable until yet, has thus been opened, which no physician or surgeon can permit himself to leave unnoticed—so much more so as Simon has also exhaustingly described all the details of his operation. We look with great anxiety for the promised second part, in which the diseases and symptoms are to be arrayed, which justify or necessitate surgical interference, and in special extirpation of the diseased kidney."—*Berlin Klinik Wochenschrift, No.* 1, 1872.

---

Treatment of Cancrum Oris.—Dr. McGreevy (*British Medical Journal*) says: "Of all the local remedies or applications I have resorted to in such cases, I have never found any application so useful or so effective as hydrochloric acid. Neither nitric acid, nitrate of silver, nor chlorate of potassa, nor any other remedy that I have ever tried or used, except hydrochloric acid, did I ever find to be of the least use to check cancrum oris. I have almost never found hydrochloric acid fail to check the progress of this dreadful disease at once, and bring on a most rapid and healthy action in the part. Nor does it cause so much pain or suffering to the little patient as one would suppose, seeing that the gangrenous spot is almost entirely without feeling at this time. This acid is easily applied to the ulcer by means of a feather or small camel-hair brush. I have cured many cases of cancrum oris by this means.—*Medical Archives.*

